# Predictive model for abdominal liposuction volume in patients with obesity using machine learning in a longitudinal multi-center study in Korea

**DOI:** 10.1038/s41598-024-79654-y

**Published:** 2024-11-30

**Authors:** Hyunji Sang, Jaeyu Park, Soeun Kim, Myeongcheol Lee, Hojae Lee, Sun-Ho Lee, Dong Keon Yon, Sang Youl Rhee

**Affiliations:** 1 Department of Endocrinology and Metabolism, Kyung Hee University Medical Center, Kyung Hee University College of Medicine, 23 Kyungheedae-ro, Dongdaemun-gu, Seoul, 02447 South Korea; 2 Center for Digital Health, Medical Science Research Institute, Kyung Hee University Medical Center, Kyung Hee University College of Medicine, Seoul, South Korea; 3 Department of Regulatory Science, Kyung Hee University, Seoul, South Korea; 4 Department of Precision Medicine, Kyung Hee University College of Medicine, Seoul, South Korea; 5 Global 365MC Hospital, Daejeon, South Korea; 6 Department of Pediatrics, Kyung Hee University College of Medicine, 23 Kyungheedae-ro, Dongdaemun-gu, Seoul, 02447 South Korea

**Keywords:** Obesity, Liposuction, Machine learning, Predictive value of tests, Body fat distribution, Surgical procedures, Outcome assessment, Clinical decision support system, Obesity, Weight management, Surgery

## Abstract

This study aimed to develop and validate a machine learning (ML)-based model for predicting liposuction volumes in patients with obesity. This study used longitudinal cohort data from 2018 to 2023 from five nationwide centers affiliated with 365MC Liposuction Hospital, the largest liposuction hospitals in Korea. Fifteen variables related to patient profiles were integrated and applied to various ML algorithms, including random forest, support vector, XGBoost, decision tree, and AdaBoost regressors. Performance evaluation employed mean absolute error (MAE), root mean square error (RMSE), and R-squared (R^2^) score. Feature importance and RMSE importance analyses were performed to compare the influence of each feature on prediction performance. A total of 9,856 were included in the final analysis. The random forest regressor model best predicted the liposuction volume (MAE, 0.197, RMSE, 0.249, R^2^, 0.792). Body fat mass and waist circumference were the most important features of the random forest regressor model (feature importance 71.55 and 13.21, RMSE importance 0.201 and 0.221, respectively). Leveraging this model, a web-based application was developed to suggest ideal liposuction volumes. These findings could be used in clinical practice to enhance decision-making and tailor surgical interventions to individual patient needs, thereby improving overall surgical efficacy and patient satisfaction.

**Supplementary Information:**

The online version contains supplementary material available at 10.1038/s41598-024-79654-y.

## Introduction

In the current cosmetic surgery landscape, liposuction remains a highly desired procedure for body contouring and fat reduction^1^. With increasing experience in liposuction, safety, patient selection, preoperative assessment, fluid management, proper techniques, and overall patient management have been emphasized and improved^2^. There has been a trend toward evidence-based liposuction based on preoperative evaluations, medications, and surgical techniques^2^.

Recent studies have increasingly focused on predicting the outcomes of liposuction surgeries, particularly concerning patient satisfaction and aesthetic results^3–6^. However, predicting the optimal volume of fat removal during liposuction remains challenging. Currently, preoperative fat assessment relies on subjective methods, such as visual inspection via photographs, waist circumference, and skin pinch measurements^7^. While recent innovations, such as ultrasound imaging^8^ and integrated web-based software^9^, have aimed to provide more precise estimations, they often lack the sophistication to account for the multifaceted nature of surgical outcomes, which are influenced by a complex interplay of patient-specific factors. Meanwhile, unlike abdominal liposuction, which focuses solely on fat removal, lipoabdominoplasty is a procedure that removes the excess skin and fat, tightens the abdominal muscle through minimally visible incisions, and corrects abdominal wall herniation^10^. These differences in surgical technique make it difficult to estimate the amount of liposuction needed for optimal outcomes. This gap highlights the need for more sophisticated predictive tools that can integrate multiple variables to enhance the accuracy of liposuction volume determination.

This study presents an innovative methodology employing machine learning (ML) to create a predictive model specifically designed to estimate liposuction volume for patients with obesity. This study explores the efficacy of ML algorithms in enhancing the precision of liposuction volume determination by analyzing data from a comprehensive longitudinal cohort study conducted at a specialized center in South Korea.

## Methods

### Patient selection and data collection

**Fig. 1:**
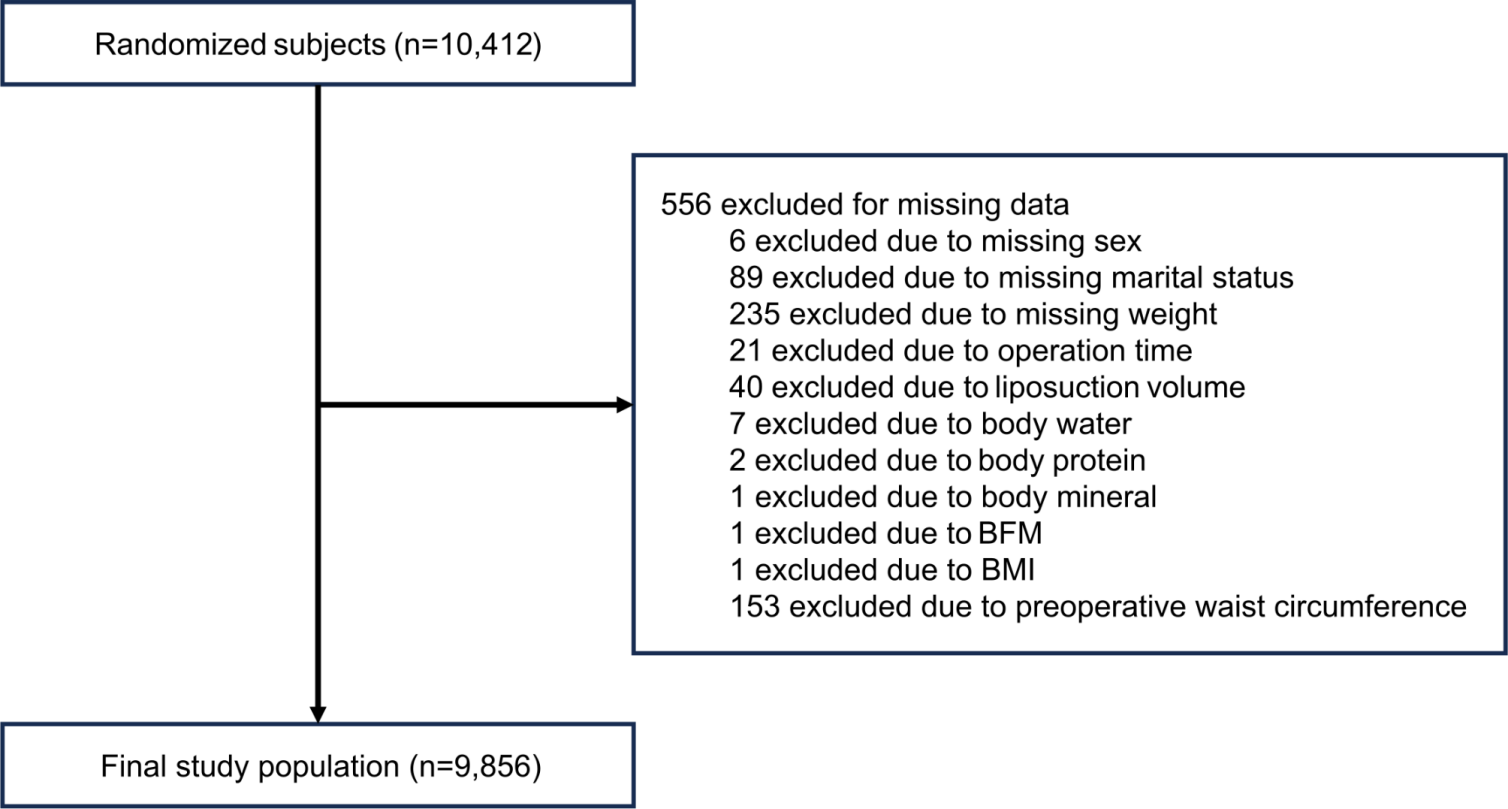
Study population. BFM, body fat mass; BMI, body mass index.

We analyzed anonymized and unidentified data from a longitudinal multicenter cohort with most cases related to liposuction in Korea (five nationwide centers affiliated with 365MC Liposuction Hospital, South Korea). We selected 10,412 subjects by random sampling from a comprehensive dataset of participants registered at five centers from August 2018 to April 2023. After omitting 556 participants due to missing data, the final sample consisted of 9,856 patients who underwent abdominal liposuction (Fig. 1).

The research methodology was approved by the Institutional Review Board of Kyung Hee University and 365MC Liposuction Hospital (Reference: KHUH 2024–04−002). The requirement for informed consent was waived by the Institutional Review Board of Kyung Hee University because de-identified data were used in the analyses. This study was conducted in accordance with the principles of the Declaration of Helsinki^11^.

### Covariates

To ensure a comprehensive representation of the patient profiles, we used 15 variables, fully leveraging the data available for our analysis. The baseline characteristics of the patients were defined by age, sex (male or female), and marital status (married, single, or divorced)^12^. The biological characteristics included weight, height, body mass index (BMI), and preoperative waist circumference. The physiological parameters, ascertained using InBody 370S (InBody Co., Seoul, South Korea), a device for bioelectrical impedance analysis (BIA)^13^, comprised body water, body protein, body minerals, body fat mass (BFM), fat-free mass (FFM), and skeletal muscle mass (SMM). Surgical variables such as operation time and liposuction volume were incorporated to provide a more comprehensive view of the dataset. The liposuction volume utilized in our analysis was derived from liposuction surgeries conducted at an obesity clinic, with the data directly entered into the database by the surgical team, akin to electronic medical record data.

### Performance metrics

Our assessment hinges on three pivotal metrics: mean absolute error (MAE), root-mean-square error (RMSE), and the R-squared (R^2^) score. MAE is the average sum of all absolute errors, whereas RMSE is a measure of the standard deviation of the evaluated deviations^14^. R-squared, also known as the coefficient of determination, ranges from 0 to 1^14^. A negative result appears if the model does not fit the algorithm, and a value closer to 1 indicates a better model^14^.

### Machine learning model implementation

Utilizing the 365MC dataset, as delineated in Fig. 1, we subjected the refined data to an array of ML algorithms. We methodically refined the dataset during the data preparation phase to satisfy analytical requirements. The key processes involved the removal of incomplete entries and the standardization of quantitative variables. To enhance the performance of the regression model, the left-skewed target variable underwent a logarithmic transformation aligned to a normal distribution. After training the model, the target variable was reverted to its original magnitude using an inverse exponential transformation, ensuring compatibility with the ML model training while maintaining the clarity of the outcomes. The optimal model was based on its minimized MAE and RMSE values approaching zero, coupled with an R^2^ score close to one^15^.

Our ML approach utilized a suite of algorithms including a random forest regressor, support vector regressor (SVR), XGBoost regressor, decision tree regressor, and AdaBoost regressor^16,17^. These algorithms were selected for their ability to handle non-linear relationships and discern intricate interactions within the data. Moreover, these models possess the advantage of handling various data types and optimizing model performance. The random forest regressor’s ability to handle many input variables and its robustness to overfitting are likely contributors to its superior performance^18^. SVR can handle linear as well as non-linear connections between the characteristics that are input and the variable that is produced^19^. XGBoost regressor effectively addresses numerous data science challenges with high speed and precision^19^. Decision tree regressor can capture non-linear correlations between the input data and the output variable^19^. AdaBoost regressor is an ensemble technique that combines weak learners by adjusting weights during training to effectively capture complex patterns and non-linear interactions, resulting in a robust and consistent predictive model^20^.

The random forest regressor, the central component of our study, was operated using the methodology depicted in Supplementary Fig. S1. The pseudocode outlines how the random forest model operates, capitalizing on its ability to capture non-linear relationships and complex interactions within the data to achieve optimal predictions.

To enhance the efficacy of these algorithms, we employed a grid search strategy that primarily focused on the R^2^ score to optimize the hyperparameters. This optimization refined the performance of the algorithm in terms of predictions.

### Feature importance and RMSE importance analysis

Feature importance is defined as the contribution of each feature to a high-accuracy space and is widely used in decision-tree-based ML methods^21^. We performed feature importance analysis on the random forest, XGBoost, decision tree, and AdaBoost regressors, which are decision-tree-based ensemble methods^22^.

To gain a more detailed perspective on the influence of individual features, we assessed the metrics based on the RMSE, as provided by the Dalex package. “RMSE importance” is calculated by systematically eliminating individual features and assessing the consequent alterations in the model’s performance^23^. It measures the extent to which the RMSE increases upon removing a specific feature. This method allows the evaluation of the impact of each feature on the predictive capability of the model^24^. Features with elevated RMSE importance values indicated their substantial influence in shaping the model’s predictions.

### Software and libraries

The dataset variables were analyzed using SAS software, version 9.4 (SAS Institute Inc., Cary, NC, USA). The analytical procedures were performed using Python (version 3.9.15) complemented by libraries such as Pandas (version 1.4.4), NumPy (version 1.21.5), Seaborn (version 0.12.0), Matplotlib (version 3.5.3), and Scikit-learn (version 1.0.2)^25,26^. The Seaborn library further aided in discerning the feature importance with the aim of identifying the key determinants in our predictions.

## Results

### Demographic characteristics

We used randomized sample data from an obesity clinic in Korea to develop and investigate an ML-based model for predicting liposuction volume in clinical patients.

The study population’s demographic characteristics were as follows: A total of 10,412 subjects participated in the dataset. After eliminating 556 subjects with missing data, the final sample consisted of 9,856 patients (Fig. 1). Table 1 illustrates the baseline characteristics of our study (mean age, 38 ± 10.4 years; female, 86.3%; mean BMI, 26 ± 4.8 kg/m^2^).

**Table 1:** Demographic characteristics of the randomized subject cohort.

Characteristics	Study set (*n* = 9,856)
Age, mean (SD)	38 (10.4)
Sex	
Male, N (%)	1354 (13.7%)
Female, N (%)	8502 (86.3%)
Marital status	
Married, N (%)	4922 (49.9%)
Single, N (%)	4833 (49.0%)
Divorced, N (%)	101 (1.0%)
Weight, kg, mean (SD)	71 (15.6)
Height, cm, mean (SD)	163 (7.0)
BMI, kg/m^2^, mean (SD)	26 (4.8)
Preoperative waist circumference, cm, mean (SD)	93 (11.2)
Body water, L, mean (SD)	33 (6.5)
Body protein, kg, mean (SD)	9 (1.8)
Body minerals, kg, mean (SD)	3 (0.6)
BFM, kg, mean (SD)	26 (9.6)
FFM, kg, mean (SD)	45 (8.9)
SMM, kg, mean (SD)	25 (5.4)
Operation time, mean (SD)	123 (44.7)
Liposuction volume, cc, mean (SD)	2632 (1484.7)

Abbreviations BFM: body fat mass; BMI: body mass index; FMM: fat-free mass; SMM: skeletal muscle mass; SD: standard deviation.

### Machine learning model results

As shown in Table 2, we evaluated the five models using MAE, RMSE, and R^2^ scores. The MAE score was the lowest in the random forest regressor (score, 0.197). The MAE scores for the other ML modules were 0.238 for SVR, 0.235 for the XGBoost regressor, 0.247 for the decision tree regressor, and 0.261 for the AdaBoost regressor.

**Table 2:** Scores of five different machine learning algorithms in our analysis.

	MAE	RMSE	*R*^2^ score
Random forest regressor	0.197	0.249	0.792
Support vector regressor	0.238	0.094	0.681
XGBoost regressor	0.235	0.303	0.690
Decision tree regressor	0.247	0.322	0.649
AdaBoost regressor	0.261	0.331	0.629

Abbreviations: MAE, mean absolute error; RMSE, root mean square error; R^2^ score, R-squared.

The model with the smallest RMSE score was the SVR (score, 0.094). The RMSE scores in the other models were random forest regressor (score, 0.249), XGBoost regressor (score, 0.303), decision tree regressor (score, 0.322), and AdaBoost regressor (score, 0.331).

The R^2^ score was closest to 1 using the random forest regressor (score, 0.792). In contrast, the SVR (score, 0.681), XGBoost regressor (score, 0.690), decision tree regressor (score, 0.649), and AdaBoost regressor (score, 0.629) had lower R^2^ scores. We found that the random forest regressor model was the optimal model for predicting the amount of liposuction in this study.

### Feature importance

**Fig. 2:**
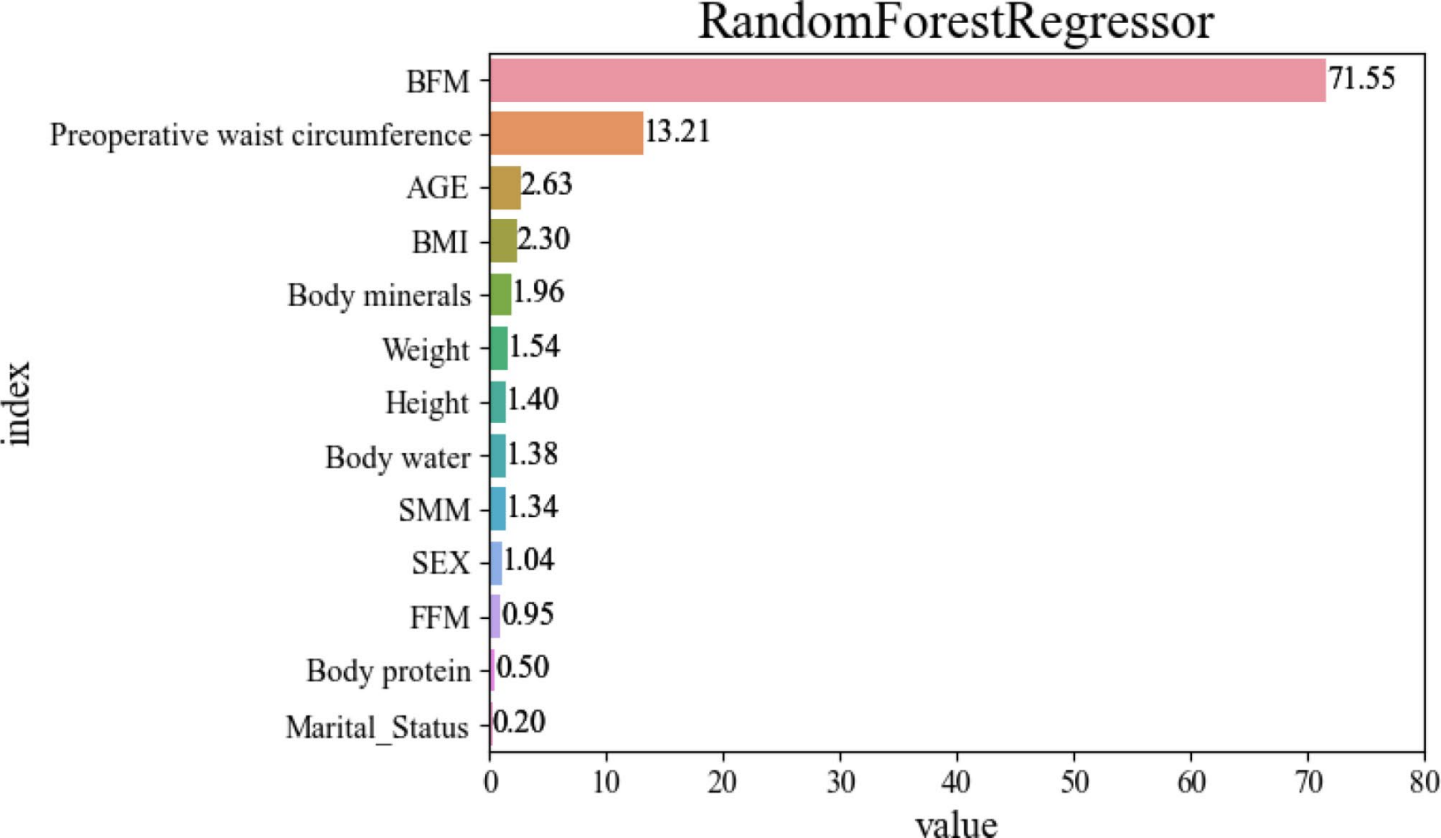
Feature importance of the random forest regressor. BFM, body fat mass; BMI, body mass index; SMM, skeletal muscle mass; FFM, fat-free mass.

Four models were used (Fig. 2 and Supplementary Fig. S2). Figure 2 shows the results of the feature importance analysis for the random forest regressor model, which had the best predictive performance among the models. BFM (71.6%) was the most influential feature in predicting the liposuction volume. The second most important feature was preoperative waist circumference (13.2%), followed by age (2.6%), BMI (2.3%), body minerals (2.0%), weight (1.5%), height (1.4%), body water (1.4%), SMM (1.3%), sex (1.0%), FFM (1.0%), body protein (0.5%), and marital status (0.2%) in descending order of importance. In Supplementary Fig. S2, similar to the random forest regressor, the XGBoost regressor, decision tree regressor, and AdaBoost regressor identified BFM as the most influential variable, followed by preoperative waist circumference.

### RMSE importance

**Fig. 3:**
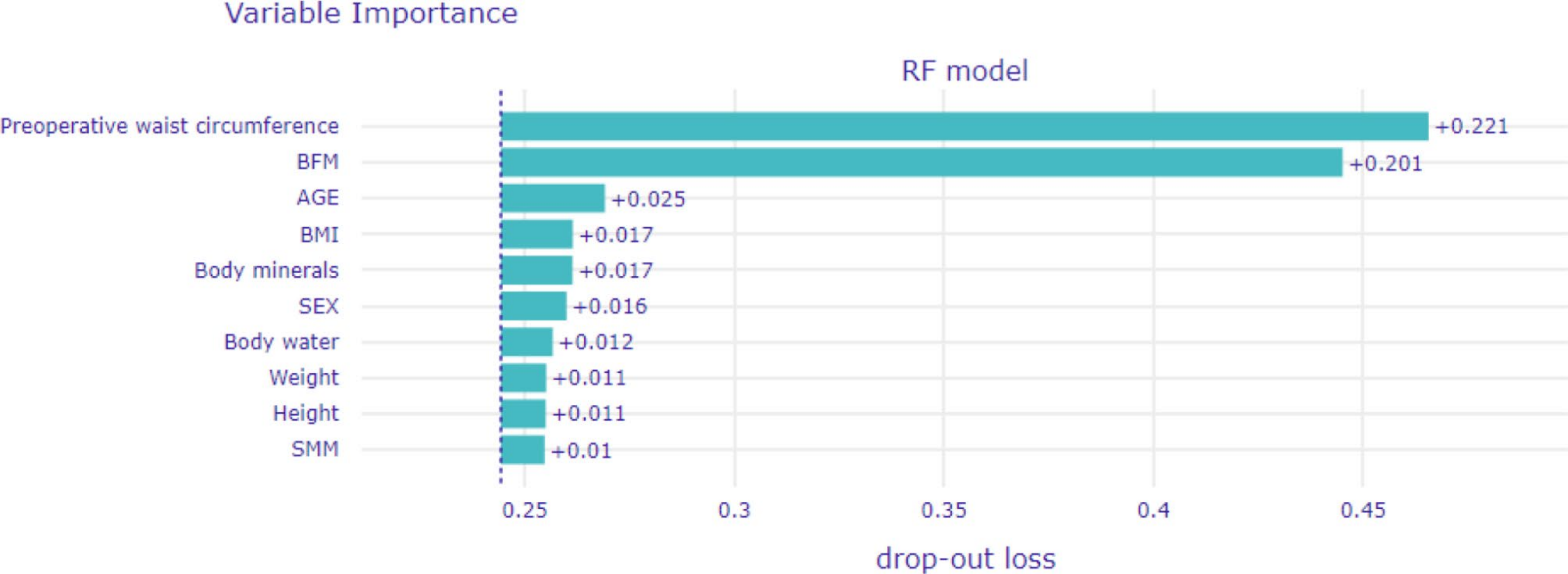
The top 10 RMSE importance of the random forest regressor. RMSE, root mean squared error; BFM, body fat mass; BMI, body mass index; SMM, skeletal muscle mass.

Figure 3 shows the top ten RMSE importance values for the random forest regressor model. The preoperative waist circumference (0.221) was the most important feature in the RMSE importance of the random forest regressor, followed by BFM (0.201), age (0.025), BMI (0.017), body minerals (0.017), sex (0.016), body water (0.012), weight (0.011), height (0.011), and SMM (0.01). In terms of RMSE importance, although the order of feature importance differed, preoperative waist circumference and BFM remained the two most significant variables.

### Development of a web-based clinical decision support system (CDSS)

**Fig. 4:**
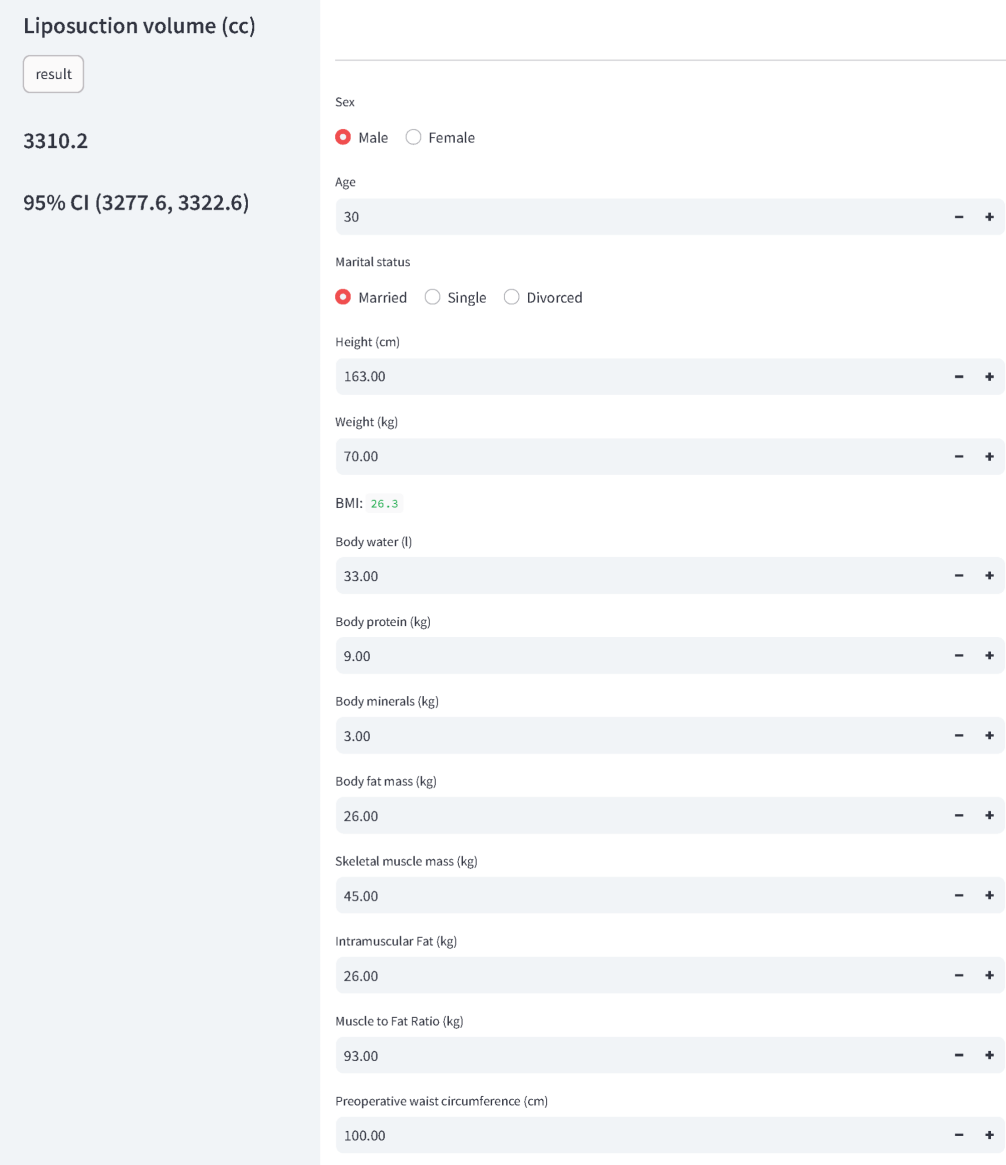
A web-based application deployed to provide liposuction amount prediction among liposuction patients. The user interface allows users to input relevant information and receive prediction results about the liposuction amount.

Based on the aforementioned ML model, we developed a web-based CDSS that predicts and suggests the optimal liposuction volume for patients at 365MC Liposuction Hospital when their preoperative variables are input. Figure 4 shows an example of the application interface. We have shared the predictive models on our laboratory website (https://liposuction.streamlit.app/). Additionally, we have implemented a simple interface to facilitate easy use of these models.

## Discussion

This study provides significant insights into the prediction of liposuction volume in patients with clinical obesity. We found that the random forest regressor model was the best predictor of the liposuction volume. While traditional liposuction outcome prediction has focused primarily on the techniques used during surgery and postoperative care and the occurrence of postoperative complications or in-hospital mortality^27–30^, our study provides a new perspective by using preoperative variables to predict liposuction volume. By incorporating a variety of demographic, physiological, and surgical variables, the ML model outperforms traditional prediction methods by combining a comprehensive dataset and rigorous ML methodology. Our study can provide clinicians with insights into the most influential factors affecting liposuction volumes and quantitative criteria for surgical success. The app interface of the CDSS has a user-friendly design that helps clinicians make quick and accurate decisions.

We analyzed a range of variables, including demographic and biological characteristics, physiological parameters of the InBody machine, and surgical variables. The population in the cohort of this study was primarily female, and their BMI and waist circumferences met the criteria for obesity and abdominal obesity in Korea^31^. BIA has a diagnostic value alongside BMI when assessing obesity^32^. In a study validating the SAT-MAP software, which uses ultrasound images to predict the amount of subcutaneous fat for liposuction, there was a high degree of agreement between the actual amount of fat removed and the predicted amount in 43 of 44 liposuction cases^8^. The findings of this study make it possible to predict liposuction volume with relative accuracy using clinical indicators and BIA metrics alone without imaging.

Using the random forest regressor model in predicting liposuction volume has significant clinical implications, particularly in enhancing surgical planning and improving patient outcomes. By accurately forecasting the optimal amount of fat to be removed based on a patient’s specific characteristics, this model enables a more individualized approach to liposuction procedures. Surgeons can use the model’s predictions to better plan the scope of the surgery, reducing the risk of over- or under-removal of fat, which is crucial for achieving desired aesthetic outcomes and minimizing the need for revision surgeries.

A key novel contribution of this study is the identification of BFM and preoperative waist circumference as the most influential predictors of liposuction volume through feature and RMSE importance analyses. Body weight, BMI, waist circumference, and BFM are objective markers for assessing the outcomes after liposuction, and these markers have been shown to decrease significantly after surgery^7,33^. However, few studies have demonstrated an association between preoperative waist circumference and BFM with liposuction volume or outcomes in abdominal liposuction. As waist circumference is a crucial indicator of abdominal obesity, it is intuitive to assume that the larger the preoperative waist circumference, the more severe the abdominal obesity, and therefore, the larger the liposuction volume. However, there are no established diagnostic criteria for assessing obesity based on BFM or percentage of body fat as measured by BIA. However, there is a positive correlation between body fat and waist circumference^34^, and body fat measurement appears to be more appropriate for assessing obesity in Caucasian populations with a BMI below 30 kg/m^2^^35^. These findings provide a deeper understanding of why waist circumference and BFM have been identified as important predictive features in liposuction volume prediction models.

Additionally, the study revealed secondary yet significant influences of factors such as age, BMI, and body composition on liposuction volume. This is somewhat consistent with other studies that identified age, sex, BMI, and surgical facility characteristics as important factors influencing liposuction-related complications^36^. These factors are consistent with the findings of the present study, which focused on patient demographics and their impact on liposuction volume estimation. Although obesity is characterized by an excess of BFM, our findings indicate that various aspects of body composition, such as body water, body minerals, and FFM, also influence liposuction volume, suggesting a more complex relationship than was previously understood^37^. Nevertheless, the results of this study indicate that various aspects of body composition, though not uniform, play a role in determining liposuction volume.

Although these findings are promising, this study has some limitations. First, reliance on data from a single South Korean clinical institution may limit the generalizability of the results. The patient population in this study, primarily consisting of Korean individuals, may have distinct demographic, cultural, or physiological characteristics that differ from populations in other regions. When applied to more diverse or international settings, these factors could influence the model’s performance. Therefore, while the model demonstrated high predictive accuracy in this dataset, its applicability to broader populations remains uncertain. In addition, the retrospective study design based on existing medical records may introduce selection bias and limit the completeness of the data. Selection bias may have influenced the findings, as the dataset was derived from a single institution with patients who voluntarily underwent liposuction. Although rigorous efforts were made to exclude patients with missing critical data, we cannot rule out the possibility that bias may have occurred due to incomplete or missing variables. There may also be complex relationships between variables such as age, sex, BMI, and body composition, but this study did not directly describe these relationships. Finally, we did not consider postoperative factors such as satisfaction or complications. This was because the main focus was to derive the estimated liposuction volume results to achieve optimal liposuction results for potential candidates based on a medical center database with high-volume surgeries. The abdominal liposuction cases included in our study were from one of the highest volume and highest success rate centers in Korea, and our ML-based model was trained on them. Therefore, we expect to minimize the number of cases in which complications, such as vascular and aesthetic compromises arising from liposuction, can be predicted. Our model aims to predict the optimal liposuction dose to assist surgeons but should be used in conjunction with surgeon expertise. Future research should examine how liposuction quantities vary in more localized body parts. They should also test the model in diverse populations, including those from different ethnic backgrounds, to confirm whether the predictive factors (e.g., BFM and waist circumference) hold the same significance in other regions. Prospective multicenter trials are needed to validate the model, with priority given to investigating postoperative factors such as patient satisfaction, aesthetic outcomes, and complications.

In conclusion, this study highlights the utility of ML models in enhancing our understanding of the factors influencing liposuction volumes in patients with obesity, thereby aiding in more informed clinical decision making. Recent advancements in the integration of ML in clinical settings suggest that these technologies can enhance precision and personalization in medical interventions, as demonstrated by our web-based CDSS^38^. This predictive model could influence future research focused on refining predictive tools for various surgical outcomes. By providing direction for personalized liposuction, these findings represent a potential paradigm shift in aesthetic and therapeutic interventions for clinical obesity. The convenient and high-performance web-based CDSS developed by the research team in this study can be integrated into clinical practice in plastic surgery and other surgical fields to enhance the precision and effectiveness of surgical interventions, paving the way for more tailored and successful patient care.

## Electronic supplementary material

Below is the link to the electronic supplementary material.

Supplementary Material 1.

## Data Availability

Restrictions apply to the availability of some or all data generated or analyzed during this study to preserve patient confidentiality or because they were used under license. The corresponding author will, on request, detail the restrictions and any conditions under which access to some data may be provided. We developed a web-based application that utilizes the ML results to predict liposuction volume in patients undergoing liposuction at an obesity clinic (website: https://liposuction.streamlit.app/). Our website and its outcomes are presented in Fig. 4.
