# Author Correction: Predictive model for abdominal liposuction volume in patients with obesity using machine learning in a longitudinal multi-center study in Korea

**DOI:** 10.1038/s41598-025-87065-w

**Published:** 2025-01-23

**Authors:** Hyunji Sang, Jaeyu Park, Soeun Kim, Myeongcheol Lee, Hojae Lee, Sun-Ho Lee, Dong Keon Yon, Sang Youl Rhee

**Affiliations:** 1 Department of Endocrinology and Metabolism, Kyung Hee University Medical Center, Kyung Hee University College of Medicine, 23 Kyungheedae-ro, Dongdaemun-gu, Seoul, 02447 South Korea; 2 Center for Digital Health, Medical Science Research Institute, Kyung Hee University Medical Center, Kyung Hee University College of Medicine, Seoul, South Korea; 3 Department of Regulatory Science, Kyung Hee University, Seoul, South Korea; 4 Department of Precision Medicine, Kyung Hee University College of Medicine, Seoul, South Korea; 5 Global 365MC Hospital, Daejeon, South Korea; 6 Department of Pediatrics, Kyung Hee University College of Medicine, 23 Kyungheedae-ro, Dongdaemun-gu, Seoul, 02447 South Korea

Correction to: *Scientific Reports* 10.1038/s41598-024-79654-y, published online 30 November 2024

The original version of this Article contained an error in the Funding section.

“This research is funded by the BK21 FOUR program of Graduate School Kyung Hee University (GS-1-JO-NON-20240382).”

now reads:

“This research was supported by the Bio&Medical Technology Development Program of the National Research Foundation (NRF) funded by the Korean government (MSIT) (No. RS-2023-00262002).”

The original Article has been corrected.

