# Supplementary material for: Predictive model for abdominal liposuction volume in patients with obesity using machine learning in a longitudinal multi-center study in Korea
Source: Sci Rep. 2024 Nov 30;14:29791. doi: 10.1038/s41598-024-79654-y (PMC11608244; doi:10.1038/s41598-024-79654-y)
Supplement: Supplementary file 1 — Supplementary Material 1. [file 41598_2024_79654_MOESM1_ESM.pdf]

Supplementary Fig. S1. Algorithmic formulas used in the random forest regressor model.

$Precondition: A\ training\ set\ S\ :$

$$= (x1, y1), \dots., (xn, yn), features\ F, and\ number\ of\ trees\ in\ forest\ B.$$

$funtion\ RANDOMFOREST(S, F)$

$H \leftarrow \emptyset$

$for\ i\ \in 1, \dots., B\ do$

$S(i) \leftarrow A\ bootstrap\ sample\ from\ S$

$hi\ \leftarrow RANDOMIZED\ TREELEARN(S(i), F)$

$H\ \leftarrow H\ \cup \{hi\}$

$end\ for$

$return\ H$

$end\ function$

$funtion\ RANDOMIZEDTREELEARN(S, F)$

$At\ each\ node:$

$f\ \leftarrow very\ small\ subset\ of\ F$

$Split\ on\ best\ feature\ in\ f$

$return\ The\ learned\ tree$

$end\ function$

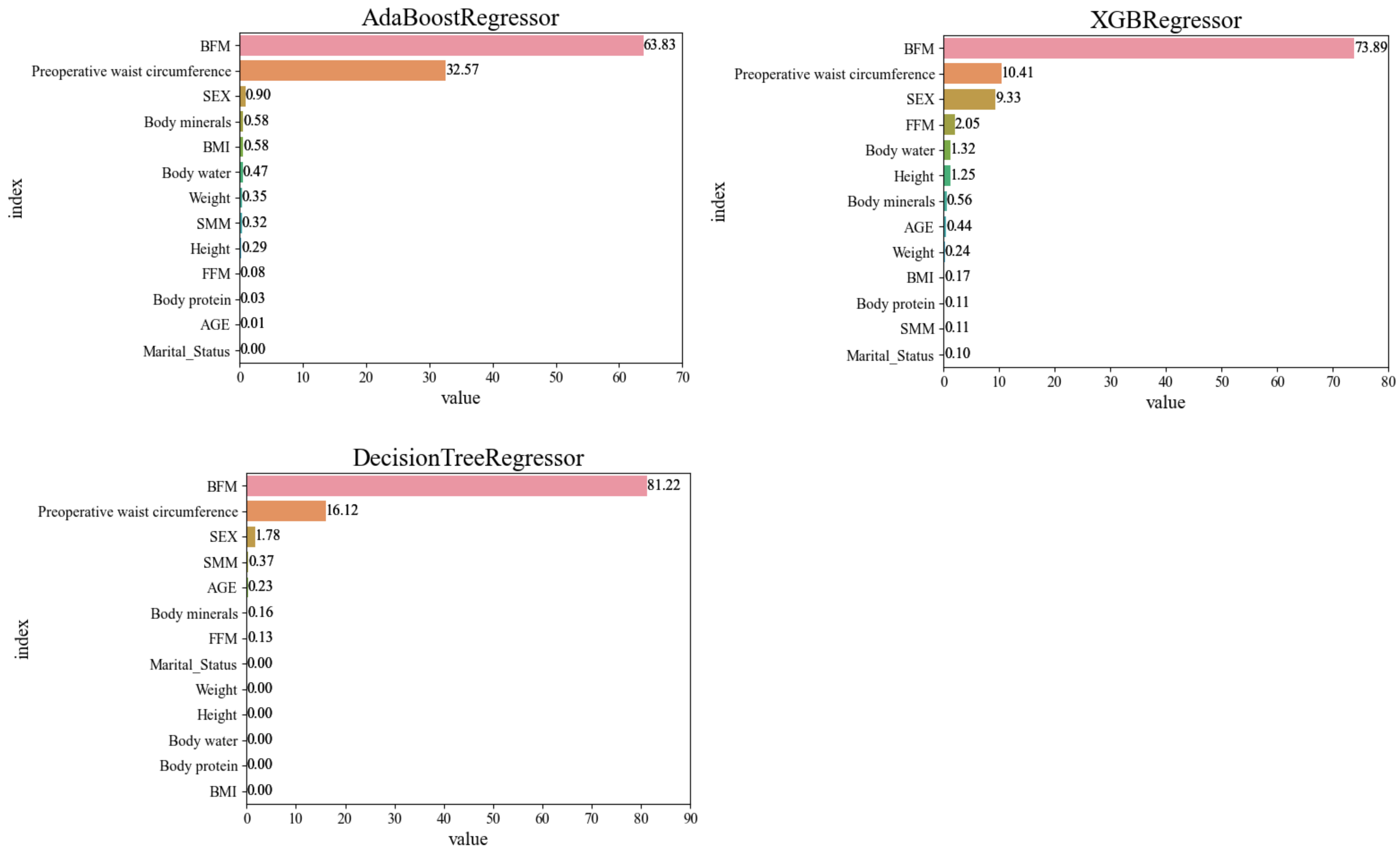

Supplementary Fig. S2. Feature importance of the AdaBoost regressor, XGBoost regressor, and decision tree regressor. XGB, XGBoost; BFM,

body fat mass; BMI, body mass index; SMM, skeletal muscle mass; FFM, fat-free mass.
